# Increased IL-17RA and IL-17RC in End-Stage COPD and the Contribution to Mast Cell Secretion of FGF-2 and VEGF

**DOI:** 10.1186/s12931-017-0534-9

**Published:** 2017-03-15

**Authors:** Abraham B. Roos, Michiko Mori, Harpreet K. Gura, Axel Lorentz, Leif Bjermer, Hans Jürgen Hoffmann, Jonas S. Erjefält, Martin R. Stampfli

**Affiliations:** 1 Department of Experimental Medical Science, Lund University, Lund, Sweden; 2 Department of Pathology and Molecular Medicine, McMaster Immunology Research Centre, McMaster University, Hamilton, ON Canada; 3 AstraZeneca R&D Gothenburg, Respiratory, Inflammation and Autoimmunity, Innovative Medicines, Pepparedsleden 1, 431 83 Mölndal, Sweden; 4 Department of Respiratory Diseases and Allergy, Aarhus University Hospital, Aarhus, Denmark; 5 Department of Clinical Medicine, Aarhus University, Aarhus, Denmark; 6 Department of Nutritional Medicine, University of Hohenheim, Stuttgart, Germany; 7 Department of Respiratory Medicine and Allergology, Lund University, Lund, Sweden; 8 Department of Medicine, Firestone Institute of Respiratory Health at St. Joseph’s Health Care, Hamilton, ON Canada

## Abstract

Mast cells are accumulated in advanced chronic obstructive pulmonary disease (COPD), and interleukin (IL)-17 signaling plays a role in disease progression. The expression, localization and functional relevance of IL-17 receptor (R)A and IL-17RC was explored in COPD by immunodetection, and functional assays.

IL-17RA and IL-17RC was increased in very severe COPD, and expressed by mast cells. Increased secretion of the pro-angiogenic basic fibroblast growth factor and vascular endothelial growth factor was observed in vitro-maintained mast cells stimulated with IL-17A. Expression of these mediators was confirmed in end-stage COPD. Thus, accumulation of mast cells in COPD may contribute to vascular remodeling.

## Letter

The expression of IL-17A is elevated in lung tissue of patients with chronic obstructive pulmonary disease (COPD), and the cytokine is linked to the pathology associated with the disease [1, 2]. In the current study, we investigated the expression of receptors activated by IL-17A (IL-17 receptor (R)A and IL-17RC), to determine the responisveness to this cytokine in COPD. We used automated immunohistochemistry to detect the expression of IL-17RA (mouse anti-IL17RA, R&D Systems, Minneapolis, MN, USA) and IL-17RC (mouse anti-IL-17RC, Atlas Antibodies, Stockholm, Sweden) in peripheral lung tissue sections obtained from a previously characterized cohort of COPD patients and relevant controls (Table 1) [3]. The local ethics committee in Lund approved the study protocol, and all subjects signed an informed consent form.

**Table 1:** Baseline demographics and clinical characteristics

	Never smokers	Smokers w/o COPD	COPD GOLD I	COPD GOLD II-III	COPD GOLD IV	p ANOVA
Subjects (n)	7	6	6	13	10	
Gender (female/male)	5/2	4/2	3/3	2/11	6/4	
Age (years)	63 ± 4.8	57 ± 3.3	67 ± 2.9	68 ± 1.9	61 ± 1.4	ns
Height (m)	1.64 ± 0.1	1.71 ± 0.1	1.74 ± 0.1	1.73 ± 0.1	1.69 ± 0.1	ns
Weight (kg)	65 ± 4.6	69 ± 4.6	69 ± 5.7	73 ± 3.1	65 ± 1.7	ns
Body mass index	24 ± 1.3	24 ± 1.0	23 ± 1.2	24 ± 1.1	23 ± 1.0	ns
Pack years	N/A	43 ± 8.2	40 ± 7.6	48 ± 3.3	41 ± 3.9	ns
Smoker/ex-smoker	N/A	4/2	3/3	5/8	0/10	
FEV1/FVC	86 ± 5.7	79 ± 2.0	67 ± 0.9	55 ± 2.8	32 ± 2.0	<0.001
%FEV1	110 ± 6.2	98 ± 6.0	87 ± 2.3	64 ± 3.1	23 ± 1.4	<0.001
Corticosteroids (yes/no/unknown)	0/7/0	0/6/0	0/6/0	2/11/0	9/0/1	
Bronchodialator (yes/no/unknown)	0/7/0	0/6/0	3/3/0	3/10/0	9/0/1	

Values are mean ± standard error of mean

*COPD* chronic obstructive pulmonary disease, *FEV*_*1*_ forced expiratory volume in 1 s, *(F)VC* (forced) vital capacity, *GOLD* Global Initiative for Chronic Obstructive Lung Disease

**Fig. 1:**
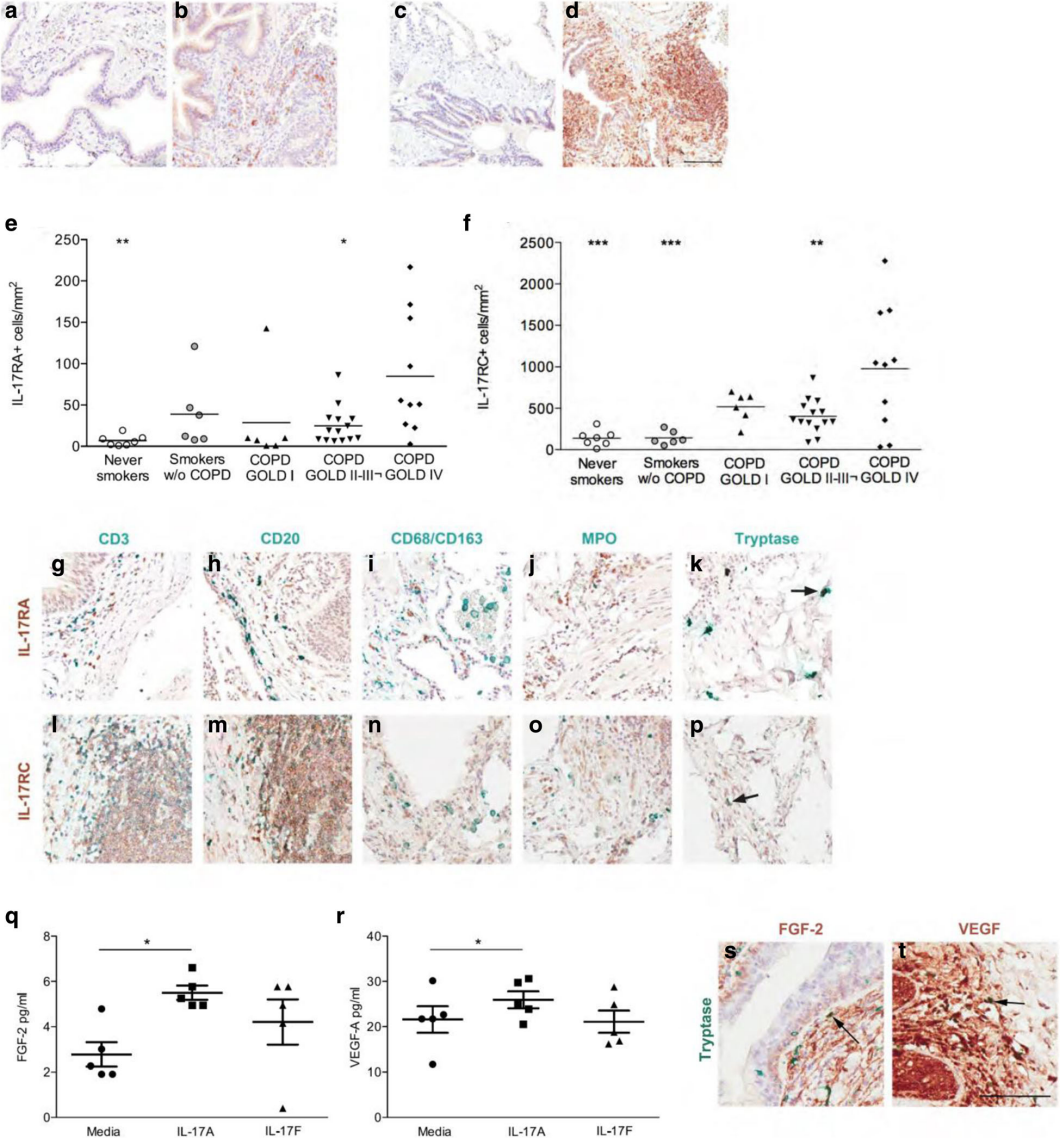
Elevated expression of IL-17RA and IL-17RC in end-stage COPD. Bright field micrographs of (**a**) interleukin (IL)-17 receptor (R)A in never smoker, (**b**) IL-17RA in COPD, (**c**) IL-17RC in never smoking non-COPD controls and (**d**) IL-17RC in COPD. Number of immunopositive cells to (**e**) interleukin (IL)-17RA and (**f**) IL-17RC in lung tissue of never smokers, smokers and patients with GOLD I-II or III-IV COPD. Horizontal line indicates mean value. One-way ANOVA and Dunnet’s test (GraphPad Prism 6, GraphPad Software, La Jolla, CA) was used to detect differences between GOLD IV COPD and all other groups. Clear circles: never smokers; grey circles: asymptomatic smokers, triangles (*up*): GOLD I COPD; triangles (*down*): GOLD II COPD; squares: GOLD III; diamonds: GOLD IV COPD. *n* = 7-18. *p < 0.05, **p < 0.01, *p < 0.001 compared to GOLD IV COPD. Co-immunohistochemical staining for (**g**-**k**) interleukin (IL)-17RA and (**l**-**p**) IL-17RC (DAB, *brown*) and cellular markers (*Vina green, blue-green*). Sections were counterstained with Mayer’s Hematoxylin. Arrows in indicate double positive cells. Scale bar: 100 μm. Expression of (**q**) FGF-2, and (**r**) VEGF in cell supernatant of stem cell-derived mast cells stimulated with 1000 ng/ml IL-17A 8 hours. Experiments were performed in triplicates and repeated twice. Bars indicate mean value, errorbars indicate SEM. *n* = 5. *p < 0.05. Light micrograph of (**s**) fibroblast growth factor (FGF)-2 (DAB; *brown*) and (**t**) vascular endothelial growth factor (VEGF) (DAB; brown) in GOLD IV COPD. Immunoreactivity was detected by DAB (*brown*) and sections were counterstained with Mayer’s Hematoxylin (*blue*). Mast cells were identified by immunodetection of tryptase (*Vena green; green-blue*). Arrows indicate FGF-2 and VEGF positive mast cells. Scale bar indicates 100 μm

IL-17RA immunoreactivity was observed within the cytoplasm of cells located within the small airway submucosa, as well as the alveolar parenchyma in both asymptomatic controls and patients with COPD (Fig. 1a-b). The airway epithelium displayed minimal immunoreactivity. Similarly, while some immunoreactivity to IL-17RC was observed in the airway epithelium, the strongest signal was observed in cells scattered within the airway submucosa and lung parenchyma (Fig. 1c-d). The number of IL-17RA+ and IL-17RC+ cells were increased specifically in very severe (GOLD IV) COPD (Fig. 1e and f), suggesting an involvement of these receptors in COPD progression. Whereas the exact mechanism underlying increased IL-17RA/IL-17RC in advanced COPD still remains unknown, it may involve the pro-inflammatory milieu documented in COPD, which may be escalated in end-stage disease.

As not only structural cells displayed immunoreactivity, co-staining techniques for different cellular markers was next performed to determine the specific cell types expressing IL-17RA and IL-17RC in the peripheral lung. In accordance with previous findings, expression was observed across different immune cell populations, including B lymphocytes (Fig. 1h and m), as well as on structural cells. Expression of both IL-17RA and IL-17RC was also detected on tryptase + mast cells (mouse anti-tryptase, Merck-Millipore, Solna, Sweden) (Fig. 1k and p). Previous studies have documented an accumulation of mast cells in lung tissue of patients with advanced COPD [3], although the role of mast cells in COPD remains elusive. We thus explored the potential functional relevance of IL-17 receptor expression on mast cells in COPD. To this end, CD133^+^ hematopoietic stem cell progenitors were isolated (*n* = 5) and differentiated into mast cells, in vitro, as described elsewhere [4]. 10^5^ mast cells were following 4 weeks maturation stimulated for 8 hours with 1000 ng IL-17A (R&D Systems, Minneapolis, MN, USA), or left unstimulated. Lipopolysaccharide (LPS, Sigma-Aldrich) was used as a positive control.

The in vitro-matured cells exhibited surface markers and functionality consistent with in vivo mast cells (i.e. expression of tryptase, chymase, c-kit and FcεR1; data not shown) [4, 5]. In addition, mRNA transcripts of both *IL17RA* and *IL17RC* were detected following in vitro-maturation (data not shown), supporting a phenotypic resemblance to pulmonary mast cells. To evaluate the potential contribution of IL-17A responsive mast cells to the pathology of COPD, we assessed the secretion of proteins associated with COPD by multiplex laser bead technology (41/11-plex Human Cytokine/Chemokine Arrays, Eve Technologies, Calgary, Canada). In line with previous documented ability of mast cells to produce pro-angiogenic factors [6, 7], IL-17A stimulated the secretion of fibroblast growth factor (FGF)-2 and vascular endothelial growth factor (VEGF) (Fig. 1q and r). Although the increases were relativiely small (2-fold increase for FGF-2 and 1.5-fold increase for VEGF), these observations demonstrate the potential role for mast cells in vascular remodeling in COPD. We used a relatively high concentration of IL-17A to activate mast cells. While the biological relevance of this concentration may be debated, the levels of IL-17A may be enriched in the pulmonary microenviroment. Furthermore, a ten fold lower dose was able to induce the secretion of FGF-2 in a pilot study (data not shown). Future studies investigating pulmonary mast cells isolated from COPD patients could provide imporant clues to the biological relevance of these initial findings.

The inflammatory mediator profile was, in contrast, unaffected by stimulation by IL-17A. The lack of pro-inflammatory responisveness of mast cells to IL-17A may be explained by earler reports of enhanced effects by other cytokines, such as TNFα, IL-1β and IL-22 [8]. Thus, future studies investigating the potential inflammatory response of mast cells in response to IL-17A would benefit from including co-stimulation, or possibly the inflammatory milieu associated with COPD.

We next investigated the immunoreactivity for FGF-2 and VEGF in lung tissue specimens from end-stage COPD patients, to confirm expression by mast cells. In support of the in vitro data establishing a potential role for mast cells in the regulation of vascular growth factors in COPD, pulmonary mast cells were positive for both FGF-2 and VEGF (Fig. 1i and j). The biological relevance of this observation needs to be examined further, in particular as structural cells exhibit marked staining intensity in pulmonary tissue.

Both increased number of pulmonary vessels and vascular area is observed in COPD [9]. Moreover, medial thickness of pulmonary vessels exhibit an inverse relation to lung function, suggesting increased vascular remodeling in advanced disease. Others have demonstrated that extra-pulmonary mast cells participate in vascular remodeling by the production of pro-angiogenic factors such as FGF-2 and VEGF [6, 7]. In addition, it was recently demonstrated that IL-17A directly induces the production of VEGF and FGF-2 by the endothelium [10].

The current data support increased IL-17 signaling in end-stage COPD, and stimulation of mast cell-derived vascular growth factors by IL-17A. Together, these findings designate a potential function for mast cells in advanced COPD, and warrant additional studies with the ultimate objective to target mast cells to reduce the morbidity associated with COPD.
